# Benchmarking the speed–accuracy tradeoff in object recognition by humans and neural networks

**DOI:** 10.1167/jov.25.1.4

**Published:** 2025-01-03

**Authors:** Ajay Subramanian, Sara Price, Omkar Kumbhar, Elena Sizikova, Najib J. Majaj, Denis G. Pelli

**Affiliations:** 1 Department of Psychology, New York University, New York, NY, USA; 2 Center for Data Science, New York University, New York, NY, USA; 3 Computer Science Department, New York University, New York, NY, USA; 4 Center for Neural Science, New York University, New York, NY, USA

**Keywords:** speed–accuracy tradeoff, deep learning, object recognition, psychophysics, benchmark

## Abstract

Active object recognition, fundamental to tasks like reading and driving, relies on the ability to make time-sensitive decisions. People exhibit a flexible tradeoff between speed and accuracy, a crucial human skill. However, current computational models struggle to incorporate time. To address this gap, we present the first dataset (with 148 observers) exploring the speed–accuracy tradeoff (SAT) in ImageNet object recognition. Participants performed a 16-way ImageNet categorization task where their responses counted only if they occurred near the time of a fixed-delay beep. Each block of trials allowed one reaction time. As expected, human accuracy increases with reaction time. We compare human performance with that of dynamic neural networks that adapt their computation to the available inference time. Time is a scarce resource for human object recognition, and finding an appropriate analog in neural networks is challenging. Networks can repeat operations by using layers, recurrent cycles, or early exits. We use the repetition count as a network's analog for time. In our analysis, the number of layers, recurrent cycles, and early exits correlates strongly with floating-point operations, making them suitable time analogs. Comparing networks and humans on SAT-fit error, category-wise correlation, and SAT-curve steepness, we find cascaded dynamic neural networks most promising in modeling human speed and accuracy. Surprisingly, convolutional recurrent networks, typically favored in human object recognition modeling, perform the worst on our benchmark.

## Introduction

A fundamental part of everyday tasks like reading and driving is recognizing objects (letters, words, signs, vehicles, and pedestrians), and there is often a large variation in the time available to do so. For instance, reaction time permitted while navigating urban traffic is much lower than when driving on limited-access highways. Thus, it is important for people to perform competently over a wide range of reaction times. When asked to recognize an object, people demonstrate higher accuracy when given more time and can also sacrifice some accuracy to respond quickly. This ability to flexibly trade off accuracy for speed is called the speed–accuracy tradeoff (SAT) and is a crucial human skill.

Deep convolutional neural networks outperform humans on popular object recognition benchmarks while also being good models for encoding in primate visual cortex (Krizhevsky, Sutskever, & Hinton, 2012; Yamins et al., 2014). There is some controversy over the use of neural networks as models of perception (Bowers et al., 2023). Engineering models can be blueprints that capture all properties of the system. However, biological modeling always has multiple levels of detail. Biologists are awed by the complexity of neural systems—from synapses to behavior. By necessity, they are reductive scientists, who come up with comprehensible ideas and evaluate how much of the bewildering biological complexity they can model (Jonas & Kording, 2017). Neural network models are generally thought of as useful models for studying the brain at the “computational” and “algorithmic” levels (Marr, 2010). Computational models describe the “design principles” of the brain, and the algorithmic models tell us how these principles could be implemented using a computer algorithm without necessarily being biologically realistic.

Independent of one’s position on modeling, it would be valuable for object recognition models to capture how human performance changes as a function of time since it is such a salient feature of human behavior. Such models are key to understanding what features are useful for recognition given different reaction times and how these useful features evolve over time. Toward this objective, we here present a large, public dataset and benchmark of the SAT in object recognition by both humans and neural networks. Our human dataset is collected using a reaction time paradigm proposed by McElree and Carrasco (1999), where observers respond at a beep that sounds at a specific time after target presentation. We measure object recognition accuracy at various reaction times by changing the beep latency across blocks.

Standard deep convolutional networks, popular in object recognition, output just a single category prediction for each input image. In order to model the temporal dimension, we (a) need networks capable of generating outputs at different *timesteps* during inference and (b) have to define *timesteps* for each of them. As candidate models, we consider an emerging class of networks that can vary the amount of computational resources they use during test-time. We chose four of these *dynamic* neural networks that use intermediate classifiers, recurrence, or parallel processing as ways to vary computational effort. One of these is a convolutional recurrent neural network (ConvRNN) that has recently been proposed as a model of the human SAT (Spoerer, Kietzmann, Mehrer, Charest, & Kriegeskorte, 2020). This network uses lateral recurrence as a way to generate outputs at different *timesteps*. Another network, CascadedNet (here referred to as CNet) (Iuzzolino, Mozer, & Bengio, 2021), implements gradual transmission in feedforward networks by taking inspiration from parallel processing in the brain and uses the number of cascaded layers as a measure of time. The other two, MSDNet (Huang et al., 2018) and SCAN (Zhang et al., 2019), are both dynamic-depth networks that were developed to improve efficiency of computer vision applications. In these, intermediate classifiers in a deep feedforward architecture correspond to different timesteps. We evaluate these networks and humans on the same images and propose three novel metrics to compare them. Our contributions are as follows:

• We tested 148 observers to present the first public dataset of controlled-time recognition of ImageNet (Russakovsky et al., 2015) images. Our participants performed 16-way categorization of images in both color and grayscale and with various amounts of noise and blur. For each condition, we tested human performance for five allowed reaction times. These data provide a benchmark for the human SAT that we hope will facilitate comparison between neural networks and humans on timed object recognition.
• We present comparable benchmarks for four dynamic neural networks: ConvRNN, CNet, MSDNet, and SCAN, which are all capable of inference-time adaptive computation.
• We compare the SAT of humans and of four dynamic neural networks using three metrics: RMSE between SAT curves, category-wise correlation, and SAT-curve steepness. Our dataset^1^ and code^2^ are publicly available.

Human object recognition is often time pressured, and finding an appropriate time-analog in neural networks is challenging. Current neural network models lack a notion of time, and neuroscience is only beginning to understand the various roles of time in biological neural networks. Our benchmark assesses how well each tested combination of network and time analog matches human behavior (i.e., accuracy vs. time curves). A floating-point operation (FLOP) can be taken as the unit of computational time. FLOPs grow with the number of repeats of operations. Different networks emphasize different kinds of repetition (e.g., layers, recurrent cycles, and readouts). For each network, in order to respect the designers’ concept, we used their repetition count as our analog for time. Toward that goal, we chose what seemed the most natural definition of timestep for each architecture: number of cycles for recurrent networks, number of exits for adaptive depth networks, and number of outputs for cascaded networks. We tested whether any of these definitions could capture the human SAT. As we will see (in Timesteps correlate with FLOPs in all five dynamic networks), in all the networks, the number of timesteps correlates strongly (*r* ⩾ 0.7) with the number of FLOPs, thus showing that the four seemingly different definitions of time that we adopted for the various network architectures are actually much alike. Unlike FLOPs, however, our measures are scale-invariant, thus allowing comparison between networks of various sizes. We encourage future work to continue this exploration, benchmarking further combinations of model and time analog, to discover which combinations adequately model human behavior.

The article is organized as follows. Related work gives an overview of related prior work. Collecting behavioral data describes our human data collection procedure and dataset. In Modeling the SAT with dynamic neural networks, we introduce the various dynamic network architectures that we evaluated on the same images that humans were tested on. Results proposes three metrics for model–human comparison and describes our findings using them. Finally, Conclusions summarizes our findings and discusses potential future work.

## Related work

### Comparing humans and neural networks

Human vision inspired early neural networks (Fukushima, 1988; Fukushima & Miyake, 1982) that incorporate some computational features of human vision (Hassabis, Kumaran, Summerfield, & Botvinick, 2017). Many properties of neural networks, such as filters (Bell & Sejnowski, 1997) and attention (Lindsay, 2020), were inspired by the human brain. Recent studies (Zador, 2019) suggest more properties that neural networks might learn from humans, and in this work, we focus on the SAT. We look at the class of networks that can vary their computational effort, a requirement to model human SAT. In machine learning literature, these models are known as dynamic neural networks (Han et al., 2021). After a single training procedure, these networks can be evaluated when forced to use different amounts of computational resources  (Bolukbasi, Wang, Dekel, & Saligrama, 2017; Chen, Li, & Bengio, 2019; Gao, Zhao, Dudziak, Mullins, & Xu, 2018; Hua, Zhou, De Sa, Zhang, & Suh, 2018; Huang et al., 2018; Li et al., 2021; Veit & Belongie, 2018; Wang, Yu, Dou, Darrell, & Gonzalez, 2018). Previous work has revealed applications of such networks in resource-sensitive applications such as analysis during autonomous driving (Yang et al., 2019) and mobile health sensors (Xu et al., 2019). Since they are the only class of neural networks that can adapt their computation to the available inference time, they are promising models of the human SAT in object recognition. We consider a representative sample of dynamic networks that use parallel processing (Iuzzolino, Mozer, & Bengio, 2021), recurrence (Spoerer et al., 2020), or intermediate classifiers (Huang et al., 2018; Zhang et al., 2019) as ways to exhibit variable computation.

### Measuring and modeling the SAT

Given more time, people generally do better. McElree and Carrasco (1999) analyzed the SAT in humans on a visual search task, in which observers tried to find a target in an array of distractors. They manipulated task difficulty by adding more distractors. Reaction time has also been studied in the context of perceptual decision-making (Basten, Biele, Heekeren, & Fiebach, 2010; Kiani, Corthell, & Shadlen, 2014; Palmer, Huk, & Shadlen, 2005; Wagenmakers, Van Der Maas, & Grasman, 2007; Wong & Wang, 2006). Previous efforts have developed drift-diffusion models (DDMs) that successfully account for the speed–accuracy tradeoff (Ratcliff & McKoon, 2008). They frame the decision-making task as a pseudo-random walk from a starting point toward one of several category boundaries as the model gains evidence. The starting point, step size, and distance between boundaries are all tunable parameters and affect both the speed and accuracy of the model (e.g., larger step sizes lead to faster convergence based on less evidence and therefore lower accuracy). Although these models accurately capture the shape of the human SAT curve, they are not image-computable (i.e., they cannot handle images as input). Mirzaei, Khaligh-Razavi, Ghodrati, Zabbah, and Ebrahimpour (2013) proposed a model to predict reaction time in response to natural images. This model is based on statistical properties of natural images and is claimed to accurately predict human reaction time by forming an entropy feature vector. Neural networks have been used to model temporal object recognition (Spoerer, McClure, & Kriegeskorte, 2017), temporal dynamics in the brain (Güçlü & van Gerven, 2017; Kietzmann et al., 2019), the ventral stream (i.e., the object recognition neural pathway in human cortex; Liao & Poggio, 2016), and temporal information (Bi & Zhou, 2020). Spoerer et al. (2020) used recurrent neural networks to model human reaction times and were the first to use a neural network as a computational model of the SAT. This work posed a binary classification problem (“animate” vs. “inanimate” objects) to human observers and networks and observed that a convolutional recurrent network explains human reaction time data well on this task. However, a binary classification task may not represent general categorization accuracy because an observer may learn to detect the difference between categories rather than actually classify images into categories. More recently, Rafiei, Shekhar, and Rahnev (2024) developed RTNet, an image-computable neural network model of the SAT that uses a DDM-like stochastic evidence accumulation process. Successive forward passes using randomly sampled weights from a Bayesian neural network (BNN) are used to update evidence toward the set of output categories. When any of the outputs reach a threshold evidence level, the network makes a prediction. In their evaluation, RTNet’s predicted reaction times correlate strongly with human data on digit classification.

## Methods

### Collecting behavioral data

We measured accuracy and reaction time for human observers performing an object recognition task on images presented with and without perturbation. Vision scientists often make use of image perturbations in probing behavior to reveal visual mechanisms. Previous work used image perturbation to reveal the channel used in visual perception for object recognition and reading (Majaj, Pelli, Kurshan, & Palomares, 2002), sources of noise within the visual system (Pelli & Farell, 1999), and visual requirements for mobility (Pelli, 1987). We seek robust similarities between humans and networks that hold up across various kinds of perturbation. We assessed the impact of adding color, blur, and noise. The results show a speed–accuracy tradeoff (Figure 4A) for all three image manipulations. In Modeling the SAT with dynamic neural networks and Results, we evaluate the ability of neural networks to model the tradeoff between processing speed and accuracy. Our experimental protocol is similar to that of McElree and Carrasco (1999) and is outlined below. Our dataset contains trial-by-trial information about observer predictions, ground-truth category, image filename, reaction time, and so on and therefore can easily be used to study timing phenomena in object recognition beyond the SAT.

**Figure 1.**
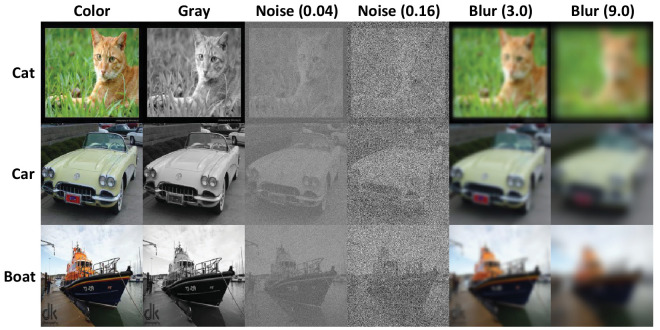
Sample ImageNet (Russakovsky et al., 2015) images shown to observers in our experiment. Rows correspond to different images, labeled with higher-level category names from 16-class ImageNet (Geirhos et al., 2018). Each column corresponds to a different image transformation used in our experiments. Noise and blur are zero-mean Gaussian, and numbers in parentheses indicate standard deviation. Noise was applied to grayscale images after reducing contrast to 20% (of original) to avoid clipping while blur was applied to the original color images.

**Figure 2.**
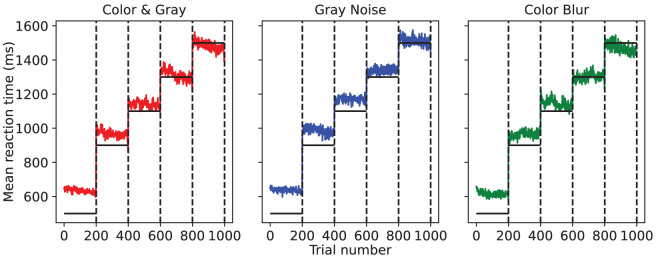
Reaction time for each timed trial in each experiment, averaged across human observers. Vertical dotted lines separate different reaction time blocks, each of which had 200 trials after discarding the first 10 used for training. Horizontal black lines indicate beep timing at which the observer was asked to respond, and colored lines denote the observer’s reaction time. Approximately 50% of observers saw trials in descending order of reaction time blocks, but we reverse their trial numbers here for the purpose of averaging and visualization.

**Figure 3.**
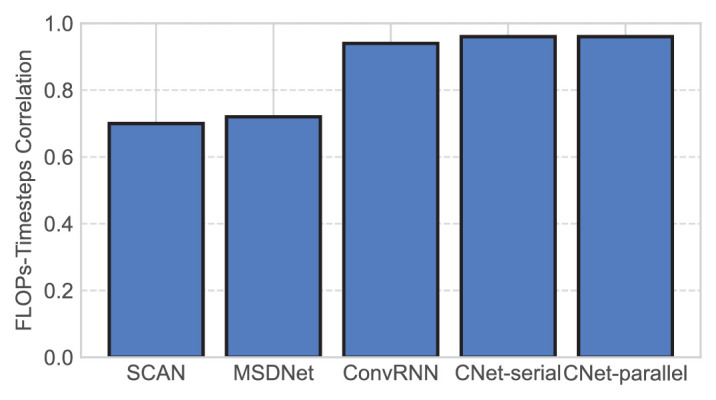
Pearson’s correlation between FLOPs and timesteps as defined for each network. Each bar represents the correlation between the array of FLOP values corresponding to each output and the timestep index (1–5).

**Figure 4.**
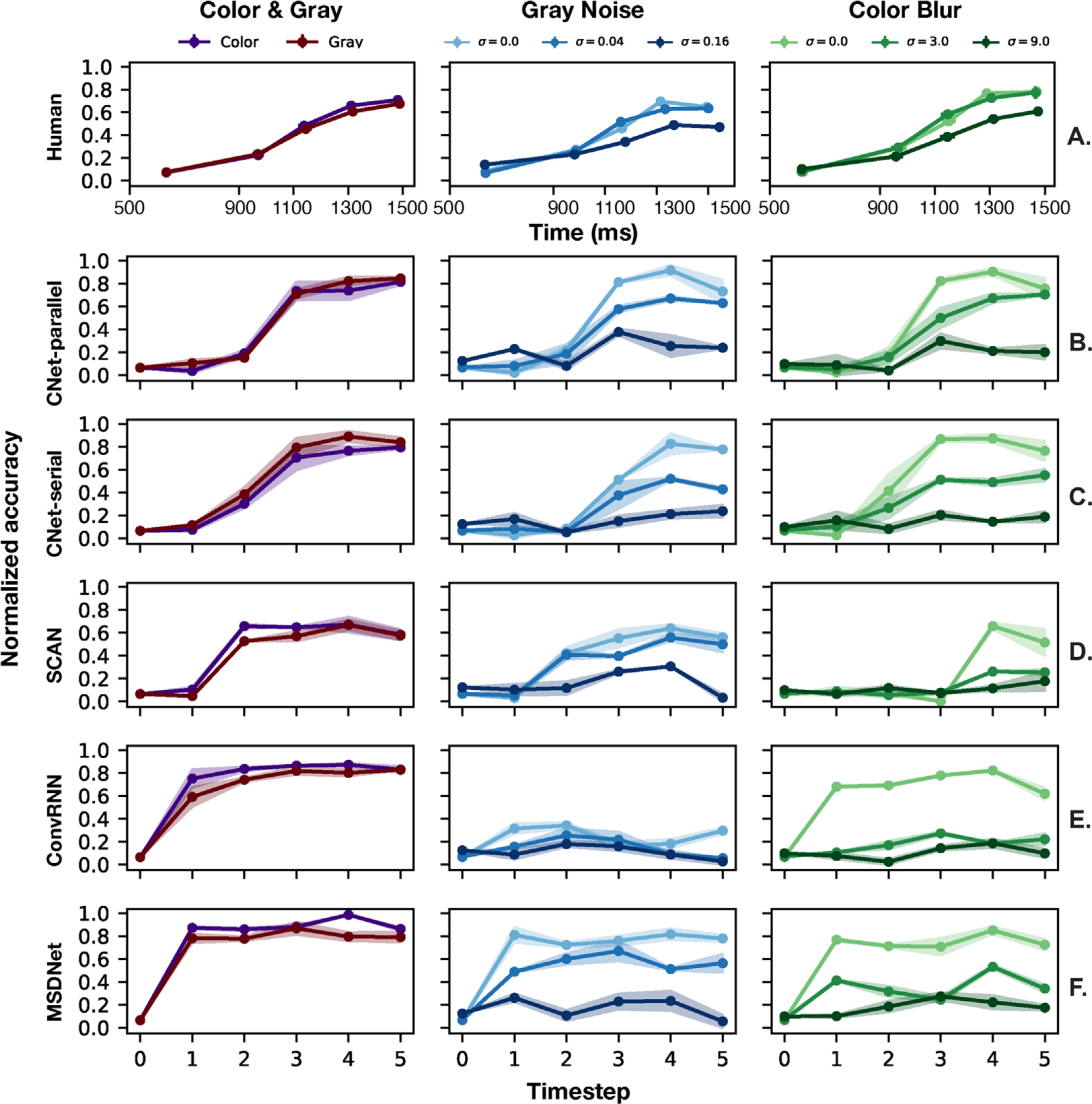
Normalized accuracy versus reaction time curves for humans and dynamic neural networks. Each point represents average accuracy normalized by human accuracy in the untimed condition and reaction time for a single reaction time block, with error bars showing standard error across both axes (barely visible because they are all very small). Each plot shows curves for all image transforms in the experiment in different colors. (**A**) Humans show a large range of accuracies over reaction times and a gradual trend, two desirable aspects that we seek to model. (**B**–**F**) For networks, the “Time (ms)” axis is replaced with “Timestep,” which is defined independently for each network (see Modeling the SAT with dynamic neural networks). Accuracy at timestep 0 is assumed to be at chance.

#### Images

In all our experiments, human observers recognized objects in images from ImageNet (Russakovsky et al., 2015), a popular natural image dataset. Since memorizing all 1,000 categories of the original ImageNet is intractable for human observers, we used 16-class ImageNet (Geirhos et al., 2018), a subset that, using the WordNet hierarchy, is labeled according to 16 higher-level categories: airplane, bear, bicycle, bird, boat, bottle, car, cat, chair, clock, dog, elephant, keyboard, knife, oven, and truck. The 16-class ImageNet has been used previously in several studies comparing human and machine object recognition primarily because numerous neural network models available publicly are pretrained on ImageNet, allowing for large-scale model–human comparison studies (Geirhos et al., 2018; Geirhos et al., 2021; Subramanian, Sizikova, Majaj, & Pelli, 2024). We use 16-class ImageNet in our article to ensure that our results can be considered in the context of results from such studies.

Due to constraints on experiment length, we used a fixed set of 1,100 randomly sampled images for all experiments. Figure 1 shows sample images under all viewing conditions used in our experiments. Images were resized from 224 × 224 to 400 × 400 pixels for optimal viewing (Pelli, 1999). Given that our experiments were conducted online, we could not measure image size and viewing distance, but we estimate the size in centimeters of the 400 × 400 pixel image to be 4 × 4 cm, subtending 4 × 4 deg, and the viewing distance (distance between observer eye and screen) to be roughly 60 cm. Since additive noise is ill-defined for color images, we converted images to grayscale for all noise experiments after also reducing contrast to 20% of original to avoid clipping at floor and ceiling. For analysis verifying that both of these modifications do not significantly degrade human performance, see Supplementary Material.

#### Observer statistics and data collection

We collected data from a total of 148 participants, recruited through Amazon MTurk (Crowston, 2012). Table 1 summarizes the statistics separately for each of our experiments. Each session (set of trials) lasted about an hour. Each observer had a normal or corrected-to-normal vision. The stimuli were presented via a JATOS experiment (Lange, Kühn, & Filevich, 2015) via worker links to each observer. Participants were paid $20 for their efforts, and the total cost of data collection was $3,860. A standard institutional review board–approved (IRB-FY2016-404 in accordance with the Declaration of Helsinki) consent form was signed before collecting the data by each observer, and demographic information was collected. Please refer to Supplementary Material for a detailed description and documentation of our dataset.

**Table 1.** Summary statistics of collected data on human observers across all three experiments. N is the number of observers in each experiment.

		Length (min.)		
Experiment	*N*	Mean	*SD*	#Trials
Color & Gray	58	43.24	13.32	1,100
Gray Noise	45	41.32	11.25	1,100
Color Blur	45	39.29	6.66	1,100

#### Experiment design

The experiment was designed to control the response time of human observers by asking them to respond in the allotted time distribution. The design was based on previous work by McElree and Carrasco (1999). In our case, three separate experiments were run for color/gray, noise, and blur. Each experiment consisted of an initial training block of 50 trials followed by five blocks of 210 trials, each corresponding to a different reaction time. The training trials were used to train observers to click one of 16 virtual buttons (corresponding to the 16 image categories) in response to the stimulus image. During the reaction time blocks, each trial involved an image being presented and required the observer to click their response at a timed beep. If the observer submitted no response within the given time, a random category was chosen as their response. Imputing missing responses with random categories biases accuracy toward chance. To verify that this bias does not severely affect our analysis, we compared random replacement with leaving out all trials where the observer failed to respond. Qualitative results using both approaches are similar. We include this analysis in Supplementary Material along with the number of missing trials in each reaction time block. An observer might be unable to respond on time either because they were unable to think of the correct category or because they did not have enough time to click on the correct response button. Psychophysically, we treat these two cases as equivalent because correct object recognition in our task is defined as correctly submitting a category response. To ensure that this is a fair assumption, we verified that the motor time (time required to click the category button when the category label was presented instead of an image) is approximately equal for all categories. Each of the five timed blocks used a different interval between the stimulus and beep: 500, 900, 1,100, 1,300, and 1,500 ms. An additional 200 ms was given after the beep for slightly delayed responses. Observer data with ≥50% of data outside a ±100 ms range of the beep were discarded. Responses within this window were accepted and used for analysis. All observers saw the same set of images within each reaction time block, in random order. Number of images from all categories was approximately equal for any given block. To avoid order effects in our results, an observer was equally likely to view the timed blocks either in ascending or descending order of reaction time. The first 10 trials of each block were discarded before analysis because they were assumed to be used by observers to adapt to the block’s beep timing.

*Note.* The SAT paradigm (Luce, 1991; McElree & Carrasco, 1999) was a major advance in tracking the improvement of accuracy with time. An alternative approach of allowing observers to respond when they feel like and then sorting into bins produces confounds that make the data hard to analyze because observers tend to take longer on harder trials. In our case, we trained observers to respond at a fixed time (different in each block), so measured accuracy is not confounded with trial-by-trial difficulty, thus making our results much easier to analyze. In many studies of the effect of timing in object recognition (Geirhos et al., 2018; Majaj, Hong, Solomon, & DiCarlo, 2015; Rajalingham et al., 2018; Tang et al., 2018), each trial's stimulus presentation and choice selection are separate steps. Various stimulus durations are reported: 100–2,000 ms (Majaj et al., 2015), 100 ms (Rajalingham et al., 2018), 25–150 ms (Tang et al., 2018), and 200 ms (Geirhos et al., 2018), after which the observers are allowed to take as much time as needed to make their selection. Another related consideration is that all models are considered visual processing models, and thus, faster response is no different from faster presentation. So why not use backward-masked time-varied presentation followed by fixed response time? Crucially for models, the image is presented for as long as they take to run inference. A human subject doing a backward-masked recognition task would be presented with an image and asked to respond when the image was no longer visible. The SAT paradigm ensures that the subject responds as the image is still present, making it an experimental setup that is more comparable to neural network models. In our experiments (the SAT paradigm), each trial was one step. The image stayed on until the observer responded. Thus, our reported reaction times include all the time between stimulus onset and response. Our observers had very little time to respond, compared to typical object recognition studies, and as a result, their accuracies are lower in our study than in others. In our case, the lowest timing threshold was specifically restricted so that the human accuracy is near chance.

During the experimental session, observers were instructed to try their best to respond at the beep, were given feedback after every trial, and were continuously presented with a trial progress counter. Clicking on the fixation cross using their mouse would invoke the next trial. This ensured that the observer fixated at the center before a trial and also that they would have to move the mouse pointer from the same location before response, thus maintaining almost constant motor time.

#### Data quality

Given that the observers had to identify complicated images perturbed with noise or blur while trying to respond exactly at the beep, the task was particularly difficult, which made it necessary to verify data quality. We checked whether the observers succeeded in responding close to the beep. Figure 2 shows the distribution of reaction time across trials in all three experiments, averaged across participants. On average, our participants responded within 100 ms of the beep across all blocks except the lowest reaction time block because 500 ms was not enough time for them to correctly categorize the presented image. Despite this, we retained this block in our analyses as the worst-case human performance. However, they displayed high consistency in reaction time across trials within all blocks.

### Modeling the SAT with dynamic neural networks

Having collected a dataset that demonstrates a strong SAT in humans (Figure 4A), we now move to using neural networks to capture this flexible, adaptive behavior. Dynamic neural networks are a class of networks that are capable of inference-time adaptive computation. Test performance can be obtained at different amounts of computation after a common training procedure. This is similar to the paradigm we used to evaluate human observers, with computational resources in networks used as an analog for human reaction time. Only a limited amount of existing work develops such dynamic architectures, in contrast to the abundance of networks available for nontemporal object recognition (Geirhos et al., 2021). We consider four network architectures that are representative of the different adaptive computation strategies and give a brief overview below. For a detailed description of architecture and training procedure, please refer to the Supplementary Material.

• **Convolutional recurrent neural network (ConvRNN)**  (Spoerer et al., 2020) exhibits temporal behavior by relying on lateral recurrent connectivity, characteristic of the primate visual system, implemented by adding layer-wise feedback connections to a feed-forward convolutional network. This model consists of several blocks of recurrent convolutional layers, followed by a readout layer to output category predictions. During inference for a given input image, the computation used by the model can be dynamically selected by running the network for a variable number of recurrent cycles. This property allows the network to respond to an input image with different numbers of forward passes, which we use as an analog for reaction time.
• **Multiscale dense network (MSDNet)**  (Huang et al., 2018) implements dynamic inference using multiple (five, in our case) intermediate classifiers from a feedforward network. Since these early exits are all at different depths, classification at each one has a different computational requirement. Since all exits use features from a common backbone network, there is an interference between the features deemed useful for classification at each depth. To resolve this problem, MSDNet proposes two architectural features: multiscale feature maps and dense connectivity (realized by using a DenseNet [Huang, Liu, van der Maaten, & Weinberger, 2017] backbone). These properties allow neurons at any layer to access features from any part of the network and at any resolution, thus diminishing the effect of the interference problem. We refer to the index of an exit as the timestep corresponding to its prediction because the five exits are approximately evenly spaced along the backbone.
• **Scalable neural network (SCAN)**  (Zhang et al., 2019), like MSDNet, implements dynamic inference using early exit classifiers from a common backbone network. Whereas MSDNet uses multiscale feature maps and dense connectivity to solve the issue of interference between early and late classifiers, SCAN uses an encoder–decoder attention mechanism in each exit network. This allows each exit to “focus” only on features relevant for classification at a specific depth. The attention network produces a binary mask, which is added to the backbone ResNet (He, Zhang, Ren, & Sun, 2016) feature map, after which a Softmax layer predicts a class label. The network uses four early exits and a final ensemble output, which uses all early exit features for prediction. Thus, for a given input, the network outputs five class predictions, each requiring a different amount of computation time/effort.
• **Cascaded neural network (CNet)**  (Iuzzolino, Mozer, & Bengio, 2021) uses skip connections and parallel processing in a ResNet (He et al., 2016) backbone to implement gradual transmission of activity. The output at the *i*th timestep uses activation at the (*i* − 1)^*th*^ ResNet block as well as partial activations from previous timesteps to simulate a cascading effect. Additionally, it utilizes a temporal difference (TD) loss whereby the target for the prediction at each timestep is the discounted sum of targets at future timesteps. The ground-truth label is used as the target for the final timestep. The version of CNet that implements both TD loss and parallel processing is henceforth referred to as “CNet-parallel.” We also consider an ablation called “CNet-serial” that also makes use of the TD loss but with no parallel processing (i.e., the output at timestep *i* is just the output when *i* − 1 blocks of the ResNet compute an activation, which goes straight to the final fully connected layer).

All networks were trained from scratch on the ImageNet dataset using the same common hyperparameters and train-validation splits to perform the aforementioned 16-way categorization task, following which they were all evaluated on the same images used in our human experiments. Since the total number of timesteps for all networks, 5, is equal to the number of reaction time values we considered, we map them linearly and use the test images for each human reaction time block to evaluate the networks at the corresponding timestep. We also include an additional sixth data point for all models, assuming chance accuracy (6.25%) at timestep 0 (i.e., when no computational resources are used). This point is only used for visualization and omitted during analysis.

### Timesteps correlate with FLOPs in all five dynamic networks

Next, we evaluate whether the time analogs used in each network correlate with FLOPs, the standard unit for computational time. For all the networks that we tested, we computed Pearson’s correlation between timesteps as defined for that network and FLOPs, with results reported in Figure 3. All networks achieved high correlation (⩾ 0.7), specifically, 0.70 for SCAN, 0.72 for MSDNet, 0.94 for ConvRNN, and 0.96 for both CNets, implying that timesteps and FLOPs have a near-linear relationship. This finding is supported by our prior results on CIFAR-10 (see Supplementary Material), which used FLOPs instead of timesteps, and found model-to-human correlations very similar to those in this article. These results suggest that FLOPs and timesteps are interchangeable for the purpose of comparing SATs, which facilitates network comparison. Unlike FLOPs, however, our timestep measures are scale-invariant and thus well suited for comparison across networks of different sizes.

## Results

Figure 4 shows the accuracy versus time or timestep curves for humans and networks. All accuracy values are expressed as a fraction of human accuracy in the untimed condition. Human accuracy gradually grows as reaction time is increased, while accounting for a large range of accuracy values. As noted by previous work using 16-class ImageNet (Geirhos et al., 2021), peak accuracy of networks drops significantly when just a small amount of noise (0.04) or blur (3.0) is introduced. But despite this drop, accuracy still increases with allowed reaction time. Also, humans perform significantly better in the untimed condition (accuracy during training trials). We hypothesize that this effect is due to more time and less cognitive load when observers are asked to respond without consideration to beep timing. Individual SAT curves for each human observer are shown in Supplementary Material. CNet-parallel and CNet-serial qualitatively capture the trends of the human curves to a high degree. They gradually increase over a large range of accuracies. SCAN’s curves increase slowly but over a much smaller range. MSDNet’s trend is very steep and shows almost no change in accuracy with time. The best networks, CNet-parallel and CNet-serial, match human performance in the zero-noise and zero-blur conditions, but once the image is degraded, we see their peak accuracy drop to as low as 20% of the human untimed accuracy, whereas peak human accuracy never drops below 40% of untimed performance. In service of a more objective analysis, we compared humans and networks using three metrics: curve-fit error, category-wise correlation, and curve steepness.

**Curve-fit error: How well do network curves match human data?** To test how well network curves match the human SAT, we computed the root-mean-squared-error (RMSE) between network accuracies and the accuracies of each human observer. Since the human and network (without the assumed timestep 0 point) data have an equal number of points (reaction times or timesteps), this was easily calculated. RMSE was found separately for each noise or blur value and then averaged. We did so because we wanted a single metric to represent how well each model captures performance across *all* values of noise or blur.

$$\begin{array}{c} \;e_{RMSE}\left(c_{1},c_{2}\right)=\frac{1}{N_{p}}\sum_{p=1}^{N_{p}}\sqrt{\frac{1}{N_{t}}\sum_{t=1}^{N_{t}}{\left(c_{1}\left[p,t\right]-c_{2}\left[p,t\right]\right)}^{2}} \end{array}$$

where *e*_*RMSE*_, the curve-fit error for two accuracy vectors *c*_1_, *c*_2_, each defined over *N*_*p*_ noise or blur perturbations and *N*_*t*_ reaction times, is the RMSE computed across time and averaged across perturbation curves. Noise and blur conditions in our experiments include regular color/gray conditions since we consider noise 0 (gray with low contrast) and blur 0 (color) cases.

**Figure 5.**
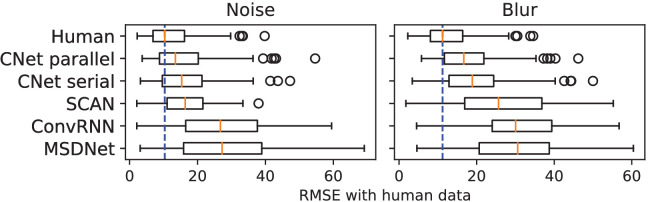
Boxplot showing RMSE of curve-fits of model data to human SAT. “Human” represents RMSE when the average human curve is fit to each human observer. Orange marker is the median RMSE. Blur dotted line is an extension of the human median. Black circles are outliers.

Figure 5 summarizes our results in a boxplot across both noise and blur experiments. We also include “Human,” which denotes the RMSE when the average human SAT curve was fit to each individual observer. We observe that across noise and blur experiments, both CNet-parallel and CNet-serial achieve very low RMSE with human data while also capturing the distribution well. SCAN’s error is low only in noise and much higher for blur, where it is similar to MSDNet and ConvRNN, which provide the least accurate fits across both experiments.

**Category-wise correlation: How similarly do humans and networks behave across object categories?** Having shown that cascaded networks, to a large extent, capture the SAT behavior in humans, we move to analyzing how the behavior of networks matches with humans across categories. Do the network and human curves resemble each other for the same categories? To understand this, we measured the category-wise correlation of each network to human data. To do so, we obtained separate SAT curves for each category, flattened them into a single vector, and then computed Spearman’s rank correlation between vectors for networks and each human observer.

**Figure 6.**
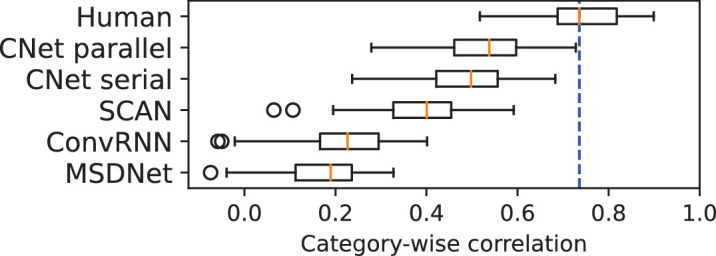
Boxplot showing category-wise Spearman’s rank correlation between curve fits of model data and human SAT in the color experiment. “Human” represents a category-wise correlation between the average human curve and each human observer. Orange marker is the median correlation. Blue dotted line is an extension of the human median. Category-wise correlation is found by computing correlation between one-dimensional vectors containing category-wise SAT curves flattened across categories.

**Figure 7.**
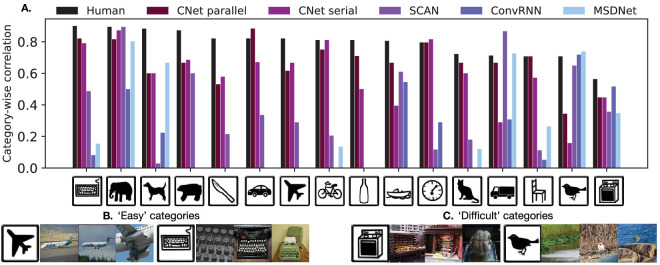
Analysis of network–human correlation across individual categories. (**A**) Barplot showing network–human correlations separately for each category. The y-axis represents median (across human observers) correlation between network and human SAT curves for a given category. Correlations were computed for all models and categories, but negative values were clipped for the sake of visualization. Icons on the x-axis are illustrations of the 16 categories from https://cocodataset.org/#explore. (**B**, **C**) Sample images from categories for which the best model, CNet-parallel, obtained high and low correlations, which are shown as “Easy” and “Difficult” categories, respectively.

Figure 6 illustrates our results as a boxplot. The CNets are the only models achieving > 0.5 correlation. However, the gap between human and CNet performance is increased relative to Figure 5. To determine a possible cause for this change, we look at Figure 7A, which shows a separate median correlation barplot for each category. We see that the CNets show a positive correlation larger than 0.5 for most categories while MSDNet, ConvRNN, and SCAN correlate poorly with several categories and sometimes also show negative correlations (clipped in figure for purpose of visualization). For the best model, CNet-parallel, “oven” and “bird” are the only cases where correlation is below 0.5. We suspect that this is due to the fact that images with birds in ImageNet often contain other objects that could confuse human observers, and the oven class is ambiguous given that microwave ovens, grills and woodfire ovens, and so on are all labeled ovens, which makes it a more difficult category for humans than CNets (Figure 7C). “Easy” categories like airplane and keyboard, on the other hand, are less ambiguous, and hence, both humans and CNet-parallel perform similarly (Figure 7B). “Easy” classes are also generally highly correlated to the average human, while “Difficult” classes show low correlation, signaling higher ambiguity. Both kinds of categories hardly affect peak network accuracy and, hence, the mismatch with humans. Figures 7B and 7C illustrate this hypothesis using randomly sampled images from categories for which CNet-parallel showed high and low correlation. Other networks, SCAN, ConvRNN, and MSDNet, are good at explaining human behavior only for a small set of categories.

**Curve steepness: How sharply does accuracy change as a function of reaction time?** A characteristic feature of the human SAT is that it is gradual (Fitts, 1966; Wickelgren, 1977). People fail gracefully as reaction time is decreased. We use mean curvature of the cumulative Weibull fit as a measure of curve steepness and compare humans with networks. We first fit the cumulative Weibull function to network and human curves, since previous work has used it to describe the SAT (Van Breukelen & Roskam, 1991). Then, we measure mean curvature, which computes on average how drastically the slope of the tangent to the curve changes between successive points. We refer to this metric as steepness. For a detailed explanation of how steepness is computed, please refer to the Supplementary Material.

**Figure 8.**
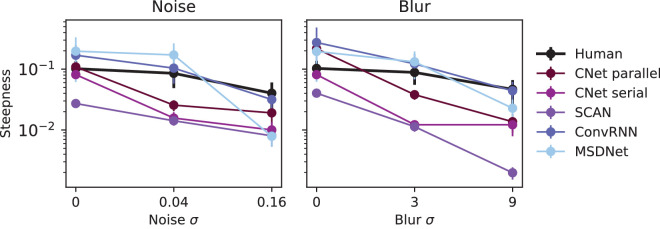
Steepness of human and network SAT curves across noise and blur conditions, shown in a log-scale. Humans show low steepness that stays almost flat across all conditions. CNets resemble human steepness range. MSDNet and SCAN show much higher and much lower steepness than humans, respectively. For the mathematical definition of steepness, refer to Results.

Figure 8 plots steepness across different noise or blur values for models and the average human, on a log scale. Our main observation is that human steepness is almost constant across all noise and blur conditions, similar to previous observations (Nachmias, 1981). This shows that people’s failure rate when time is decreased is independent of task difficulty. MSDNet is, on average, steeper, changing very little between low noise or blur values but significantly between mid-high values. This is expected since the MSDNet curves in Figure 4E show a very steep rise for low noise or blur conditions. SCAN shows significantly lower steepness than humans in both noise and blur. This is due to the fact that SCAN covers a small range of accuracies by increasing very gradually. In general, none of the networks adequately capture the flatness of the human psychometric curve.

**Figure 9.**
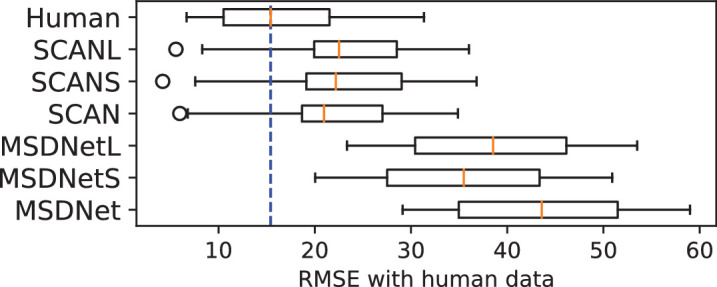
Boxplot showing RMSE between model and human data for size-varied versions of SCAN and MSDNet. Data shown are for the Gray image condition. SCANS, MSDNetS and SCANL, MSDNetL are smaller and larger versions of SCAN and MSDNet, respectively. “Human” represents RMSE when the average human curve is fit to each human observer. Orange marker is median RMSE. Blur dotted line is an extension of the human median. Black circles are outliers.

**Size effects: How does correlation with human data change with network size?** One could argue that the fast saturation of the SAT curve and therefore low model–human RMSE, observed in SCAN and MSDNet, arises because of large network size and not their architectures. To test this, we repeated the same experiments and analysis as before for smaller and larger versions of our networks. We replaced the ResNet-18 backbone of SCAN with ResNet-9 and ResNet-34 to yield SCANS and SCANL, respectively. Similar variations were applied to the MSDNet architecture to give MSDNetS and MSDNetL. Figure 9 shows the RMSE computed with human data for all variations of these networks. We see that these changes to network size do not significantly affect results.

## Discussion

The speed–accuracy tradeoff is an important feature of human performance that is difficult to explain with current computational models of object recognition. People perform better when given more time but can also respond quickly when needed, with some loss in accuracy. Trained deep convolutional neural networks, like people, can recognize objects from images. These networks, popular in computer vision, also successfully model both object recognition behavior and neural activity in humans and nonhuman primates (Geirhos et al., 2021; Yamins et al., 2014) and can predict aspects of biological vision beyond performance, such as adversarial examples (Guo et al., 2022), and object representation topography (Doshi & Konkle, 2022). However, these networks still lack a definition of time, which is a critical dimension of object recognition and decision-making by humans. Models of human reaction time generally include fixed delays (e.g., about 70 ms for the retina and several hundred milliseconds to plan and execute the keyboard response, depending on number of alternatives, distance the hand must travel, and required spatial precision. Those delays are independent of visual task difficulty. Assuming simple proportionality would not cope with those fixed delays. Our modeling deals with this by allowing an unconstrained linear mapping (speed and delay, not just speed) between the network measure (timesteps) and the human reaction time in milliseconds. Standard deep networks are all-or-none; they can either respond to a task using all of their parameters or not respond at all, whereas people adapt to various time constraints and fail gracefully. To bridge the gap between neural networks and biological vision, it is therefore essential that neural networks explain this time-dependent behavior. As a step toward that goal, we use dynamic networks, a special class of deep networks capable of flexibly varying their computational resources. They do so by using one of several possible strategies on top of a standard convolutional network backbone. In our analysis, we consider a representative sample of these strategies: early exits, recurrence, and parallel processing. Previous work in neuroscience supports recurrence (Kar, Kubilius, Schmidt, Issa, & DiCarlo, 2019) and parallel/distributed processing (Kugele, Pfeil, Pfeiffer, & Chicca, 2020) as viable representations of time in biological networks. Rafiei, Shekhar, and Rahnev (2024) performed a different but relevant analysis of some of the networks we tested (CNet-parallel, MSDNet, ConvRNN) as models of human SAT on a MNIST-digit categorization task. While we observe variations in accuracy at fixed reaction time blocks, they allowed the model to decide when to respond. Across different measures, they saw that while CNet does account well for human accuracy and reaction time data, it is outperformed by RTNet, a stochastic evidence-accumulation neural network model.

While timing of object recognition has been explored (Thorpe, Fize, & Marlot, 1996), its speed–accuracy tradeoff has not. It is a crucial characteristic of biological object recognition, so we here present a benchmark of humans and neural networks (Geirhos et al., 2021). By collecting data that include both accuracy and time, we reveal properties that allow us to better select models. Our dataset is enhanced by including several kinds of image degradation. Existing work has observed that easy and difficult images (as defined by categorization performance) are processed for different amounts of time physiologically (Kar et al., 2019). Image degradation allows control over an image’s difficulty, so it might be useful to study SAT with image degradation physiologically (Okazawa, Hatch, Mancoo, Machens, & Kiani, 2021; Oleskiw, Pasupathy, & Bair, 2014).

What architectural features yield better models of SAT? Among the networks we tested, Cascaded networks were the only ones to make use of an SAT-specific loss function: They introduced variable penalties on the loss function used to train each output. By accepting more error for fast predictions, they explicitly induce a more gradual increase in performance with respect to time. On the other hand, worse-performing networks like MSDNet, SCAN, and ConvRNN are not constrained to show an SAT by their objective function and instead rely only on lower computation to simulate worse performance at lower time durations. Whether there is any correspondence between features of cascaded networks and the brain remains an open question. Also, how do the features and representations used by the brain change with time (Holcombe, 2009)?

Most perceptual tasks exhibit the speed–accuracy tradeoff, but some cognitive tasks do not. Domingue et al. (2022) report data for 19 cognitive tasks in which accuracy drops with more time, for at least part of the range. Eleven of these tasks enforce an upper limit on response time and mostly (9 of 11) show a sharp drop just before the time limit, indicating that people are less accurate under high time pressure. Some other tasks (chess, working memory, set) can either be solved instantly or not at all because they rely on memory or intuition. Thus, to account for time-based behavior beyond the classic SAT, variables like memory and stress should be captured. By accounting for the temporal dimension, dynamic networks benchmarked in our article are a useful starting point.

Dyslexia is a highly prevalent but poorly understood slow reading condition. Although there exist various theories for it, they lack computational models that explicitly incorporate time (Ramus et al., 2003) (but see Legge, Klitz, & Tjan, 1997). Object recognition models with adaptive inference time could serve as valuable computational models for simulating both slow and fast reading processes in dyslexia research. These models allow researchers to investigate how factors such as word familiarity, contextual cues, and individual differences in processing speed contribute to reading performance in both typical and atypical readers (Donnarumma, Frosolone, & Pezzulo, 2023).

## Conclusions

We collected a large SAT dataset of humans recognizing ImageNet images presented in color or grayscale with various levels of noise and blur. In these data, people are more accurate when given more time, across all conditions, demonstrating a large range of accuracy (6.25% – 75%) and a smooth monotonic rise with time. We evaluated the behavior of neural networks as models of the human SAT. In order to compare network and human performance, we rely on three metrics: curve-fit error, category-wise correlation, and curve steepness, which allow for a robust analysis. We determined that cascaded dynamic neural networks (CNets) can, to a high degree, capture the human SAT across all noise and blur conditions and also have high category-wise correlation to human SAT. The human curve’s 0.1 log–log steepness is conserved across all noise and blur conditions. Machines generally have higher steepness, which is not conserved, varying 2- to 100-fold. One network CNet-parallel’s steepness resembled human steepness.

## Supplementary Material

Supplement 1

## Footnotes

1 See https://osf.io/2cpmb/ for dataset.

2 See https://github.com/ajaysub110/satbench for code.
