# Supplementary material for: Benchmarking the speed–accuracy tradeoff in object recognition by humans and neural networks
Source: J Vis. 2025 Jan 3;25(1):4. doi: 10.1167/jov.25.1.4 (PMC11706240; doi:10.1167/jov.25.1.4)
Supplement: Supplement 1 [file jovi-25-1-4_s001.pdf]

Supplementary Material

Appendix A Behavioral data collection

Figure A1 illustrates the sequence of screens in our psychophysics experiment along with sample screenshots for each screen. Raw data of human accuracies vs reaction time, resulting from our experiments is shown in Figure B2. Our dataset was collected via Amazon Mechanical Turk (Crowston, 2012) wherein each participant was paid \$20 for approximately an hour. 193 observers participated in our experiments. Thus, the total cost of data collection was \$3860. Data from 45 participants were discarded because more than 50% of their responses were outside the required reaction time window of ±100 ms from the beep. Therefore, we present all our results on the remaining 148 participants.

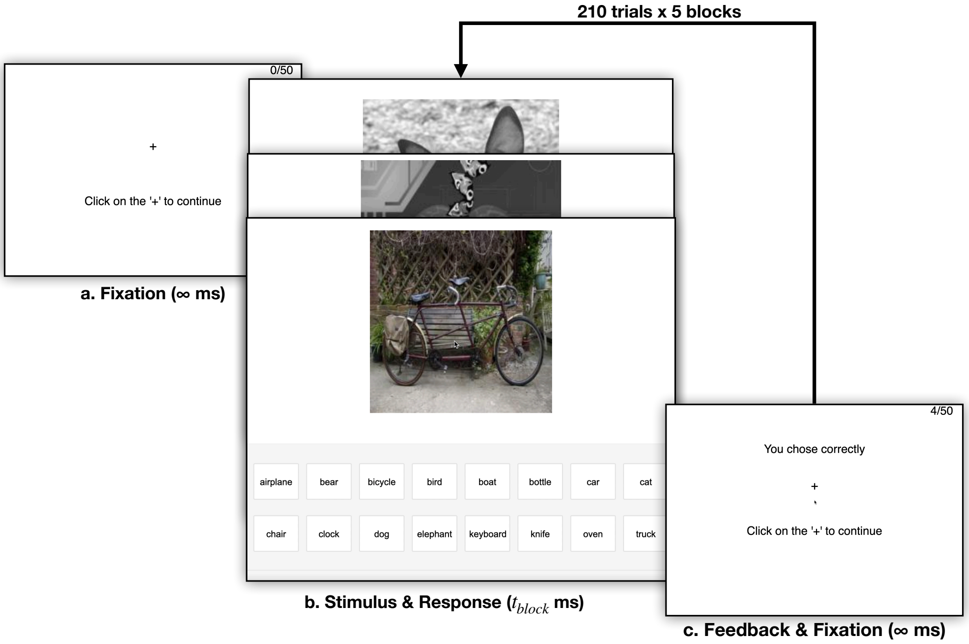

Fig. A1 *Sequence of screens in each trial of our psychophysical experiment.* **a.** Each block in our experiments begins with a fixation screen which prompts the observer to click on a central cross. This is done to ensure that the observer fixates at the center of the image at the beginning of each trial. Clicking the cross presents the **b.** stimulus & response screen. An image and 16 category buttons below it are displayed. The observer is asked to select a category when a beep sounds at t_{block} ms. **c.** Then, the next screen gives feedback and solicits fixation for the next trial.

Appendix B Contrast adjustment for noise experiments

In our experiments evaluating humans and networks on noisy images, we perturbed images with Gaussian noise of zero mean and various standard deviation values. Since noise is additive and pixel values can only lie in a finite range (0.0-1.0), large perturbations to images could result in clipping which in-effect changes the noise distribution to salt-pepper. Thus, to make sure that our noise is Gaussian, we lower the contrast of the image to 20% of the original before adding noise. Before doing so however, we have to verify that lower contrast does not deteriorate human performance, we ran an additional experiment testing people on original and 20% contrast images. Results are shown in Figure B3. We see no significant effect of lowering contrast on accuracy.

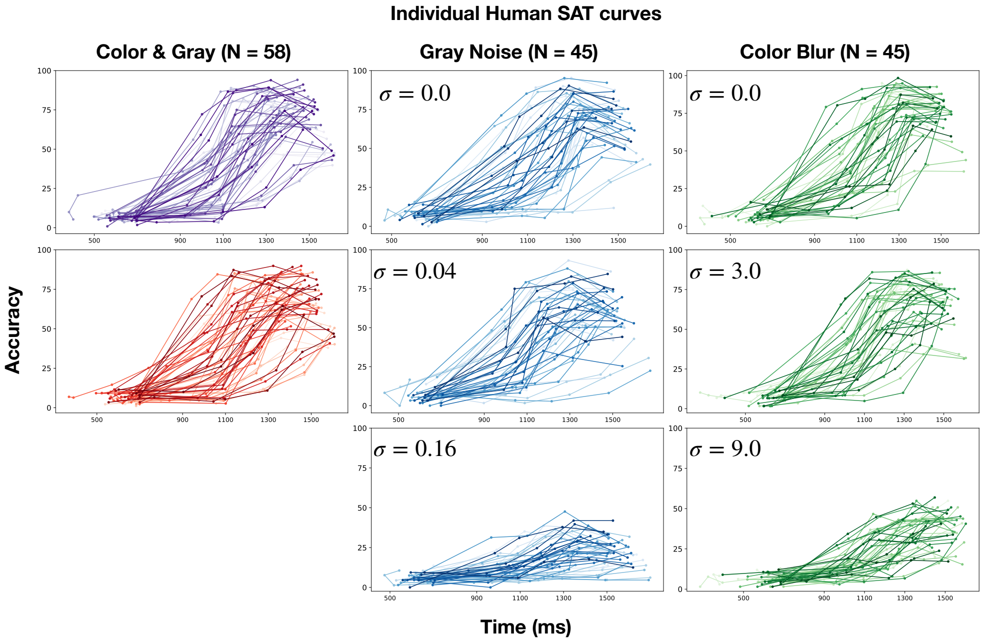

Fig. B2 *SAT curves for each human participant across all experimental conditions.* The curves are monotonic, showing the familiar sigmoidal increase of accuracy with time.

Appendix C Training and evaluation details

All networks were trained on the ImageNet training set on 16-way categorization and evaluated on the same data used for humans. For all networks, we used data augmentation during training based on standard techniques mentioned in (Huang et al., 2018): images are horizontally flipped with probability 0.5, normalization based on channel means and standard deviation is also done.

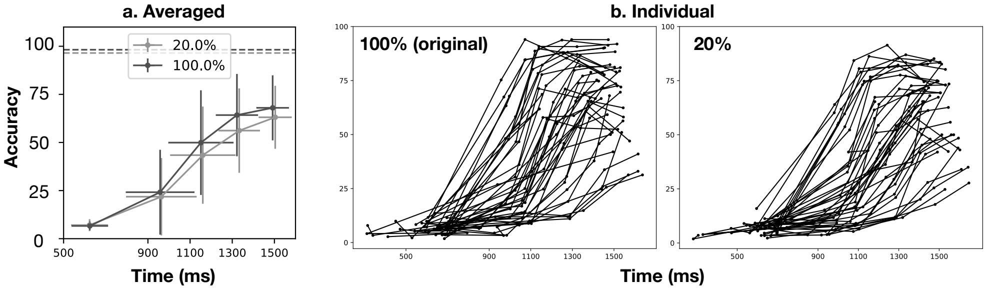

Fig. B3 *SAT curves when original (100%) and low contrast (20% of original) grayscale images were presented.* **a.** Averaged data for original and low-contrast conditions. Dotted lines denote untimed condition. **b.** Participant-wise SAT curves for original and low-contrast conditions. We notice a large variance in SAT curves but even the participants that required more time reached near-100% in the untimed condition.

C.1 Cascaded neural network (CNet)

For both serial and parallel CNet architectures, cross-entropy loss augmented with temporal difference (TD) learning is computed on a modified target y_t at each timestep t.

$$y_t = (1-\lambda)\Big[\sum_{i=1}^{T-t} \lambda^{i-1}\hat{y}_{t+i}\Big] + \lambda^{T-t} y_{true}$$

where $\lambda \in [0,1]$ is a hyperparameter that governs weighting between the true target and future timestep predictions (Iuzzolino et al., 2021). The model is trained over 120 epochs with batch size 128 and uses a Stochastic Gradient Descent (SGD) optimizer with Nesterov momentum 0.9 and weight decay 0.0005. We apply a multi-step decaying learning rate scheduled every 30 epochs with a starting rate of 0.01 and decay factor 0.2.

C.2 Convolutional recurrent neural network (ConvRNN)

To prevent overfitting, the model was initialized with pre-trained ImageNet (Russakovsky et al., 2015) weights and all layers before fully connected layers were frozen for subsequent training. The network was trained to optimize cross-entropy loss over classification targets using Adam optimizer with learning rate 0.005 and epsilon parameter 0.1. L2 regularization was applied throughout training with coefficient of 10^{-6}. The model was trained for 100 epochs with a batch size of 128.

C.3 Multi-scale dense network (MSDNet)

During training, MSDNet uses a cumulative cross-entropy classification loss computed over all early exits. The model is trained for 300 epochs and uses a Stochastic Gradient Descent (SGD) optimizer with a learning rate of 0.1 and batch size of 64. During evaluation, 5 approximately equally spaced exits are used.

C.4 Scalable neural network (SCAN)

During training, a loss function that combines a cross-entropy term (for classification) and a self-distillation term is computed and summed over all exits. The self-distillation helps improve accuracy by encouraging a low KL-divergence between the exit outputs and final output distributions, and is controlled using a self-distillation coefficient. In our experiments, SGD with a fixed learning rate of 0.1 and momentum factor of 0.9 is used to optimize network parameters. We use the default self-distillation coefficient of 0.5 and with a batch size of 128, for 50 epochs. During evaluation, 5 approximately equally spaced exits are used.

Appendix D Evaluation metrics

D.1 Category-wise SAT curves

Figure D4 shows category-wise accuracy-time curves for the average human and all networks. In humans, the difference between category accuracies emerges only for large reaction time values. This is contrary to our expectation that easier categories would peak earlier than more difficult ones. Qualitatively, CNet-parallel captures the general trend of the human curves the best and CNet-serial follows closely behind. ConvRNN and MSDNet curves peak very early on and them along with SCAN show much larger differences between categories at early timesteps than humans do.

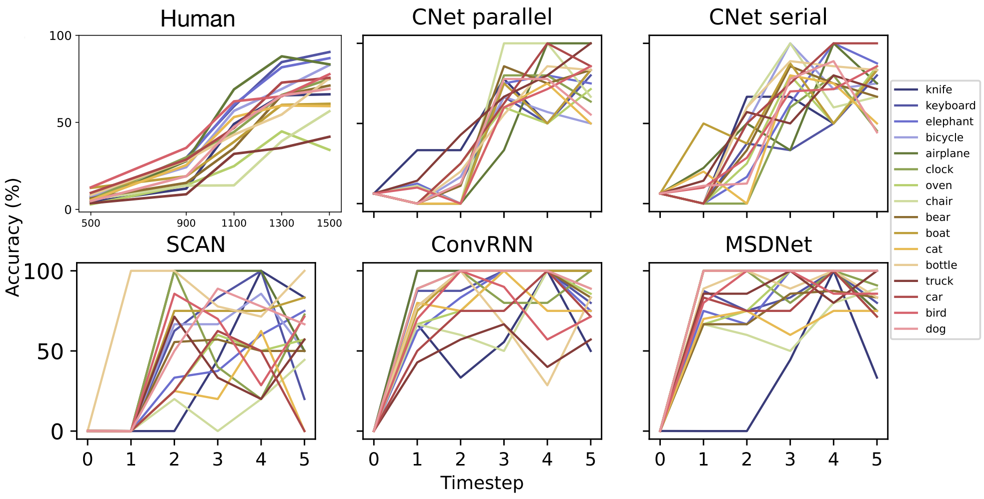

Fig. D4 *Category-wise accuracy-time curves for average human and all networks.* CNets, like humans, peak later and show similar behavior across all categories.

D.2 Curve steepness

We proposed the steepness metric as a way to compare humans and networks on their ability to gracefully fail as time is decreased. We use mean curvature as a measure of steepness. A cumulative Weibull function $w(x)$ is first fit to the accuracy-time data points.

$$w(t) = 1 - e^{-(t/\lambda)^k}$$

where t is the input to the function (reaction time, in our case) and λ, k are parameters to be fit. We use non-linear least squares to find the function that best fits each curve and then sample 20 equally spaced points for each. Finally, curvature κ is found as shown below.

Speed is computed as the slope of the tangent to the curve at each sampled point. It is also the magnitude of the velocity vector (gradient of curve). This value will be used later on.

$$\text{Speed} = \frac{ds}{dt} = |\mathbf{v}(t)| = \sqrt{(x')^2 + (y')^2}$$

The tangent vector $\mathbf{T}$ to the curve is found by dividing the velocity vector $\mathbf{v}$ by speed.

$$\mathbf{v} = \frac{ds}{dt}\mathbf{T}, \quad \mathbf{T} = \frac{\mathbf{v}}{ds/dt}$$

Then, acceleration $\mathbf{a}(t)$ or the rate of change of the tangent's slope, is the derivative of velocity and can be expressed as:

$$\mathbf{a}(t) = \frac{d^2 s}{dt^2}\mathbf{T} + \kappa \left(\frac{ds}{dt}\right)^2$$

using which the curvature κ can be obtained for plane curves as:

$$\kappa = \frac{|x''y' - x'y''|}{\left((x')^2 + (y')^2\right)^{3/2}}$$

The above equations are implemented in code as follows.

```
import numpy as np

def find_curvature(points):
        # calculate velocity
        x_t = np.gradient(points[:,0])
        y_t = np.gradient(points[:,1])
        vel = np.array([ [x_t[i], y_t[i]] for i in range(x_t.size)])

        # compute speed
        speed = np.sqrt(x_t * x_t + y_t * y_t)
```

```
# compute tangent
tangent = np.array([1/speed] * 2).transpose() * vel

# find curvature
ss_t = np.gradient(speed)
xx_t = np.gradient(x_t)
yy_t = np.gradient(y_t)
curvature_val = np.abs(
xx_t * y_t - x_t * yy_t
) / (x_t * x_t + y_t * y_t)**1.5

return np.mean(curvature_val), np.std(
curvature_val
)/np.sqrt(len(curvature_val))
```

Appendix E Accounting for the possibility of motion bias in human data

Since our measure of reaction time for human observers includes the time required to move the mouse, we had to test for the possibility of motion-induced bias in our reaction time measurements. We did so in two ways. Firstly, on the blank screen before each trial, the observer was asked to click on the central fixation cross to ensure that mouse motion before each response always started from the same position on the screen. This eliminates any variance in RT measurements due to starting position. Secondly, we ran a baseline experiment where observers saw a category name instead of an image and had to click on the button containing the same name. This was done to measure the pure motor time required to move from the fixation cross to any category button. We observed no significant difference between categories on this task which confirmed that our data was free of motion bias.

Appendix F Correlation with human data is also network-size agnostic on CIFAR-10

As support to our results in the main paper showing that network size does not affect RMS errors between network and human data, Figure F5 shows similar results (Pearson correlation instead of RMSE) for the same task and networks on the CIFAR-10 (Krizhevsky, 2009) image dataset. SCAN-R18 is the original SCAN model that uses a ResNet-18 backbone. SCAN-R34 and SCAN-R9 are modifications that use larger (ResNet-34) and smaller (ResNet-9) backbones respectively. Similarly, MSDNet-S and MSDNet-L are smaller and larger versions of MSDNet (MSDNet-M). There is no significant difference between correlations MSDNets or SCANs of different sizes.

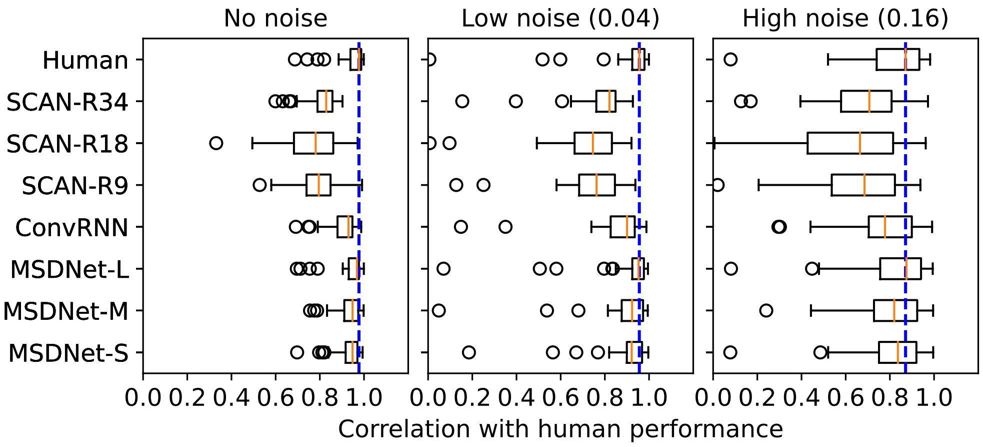

Fig. F5 *Correlations of SAT curves to human data, computed for networks of various sizes for the noisy image condition.* Orange line indicates median measurement. Blue dotted line is an extension of the human median.

Appendix G Handling missing responses

Given that our psychophysical paradigm relied on people being able to respond within some interval of a timed beep, there was a fraction of trials in our experiments where the observer failed to respond on time, leading to a missing categorization response. In the main paper analyses, we replaced missing responses with randomly sampled categories. While this allows us to perform our analyses without sacrificing the dataset size, it biases all accuracy measurements towards chance ($\frac{100}{16}\%$). To make sure this doesn't severely affect our results in the main paper, we performed all our analyses simply leaving out missing-response trials and found that all our qualitative results remain the same: CNets are still the best model across all metrics. Figure G6 presents fraction of missing responses per timing block along with RMSE curvefit-error boxplot when analysis left out problematic trials.

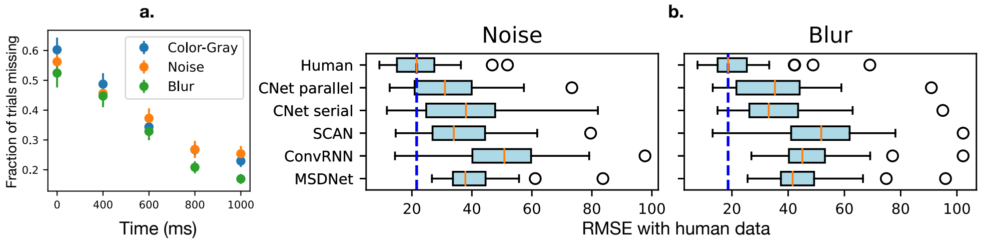

Fig. G6 *Handling missing responses.* **a.** Fraction of missing trials across time blocks. **b.** RMSE curve-fit errors when missing-response trials were left out.

Appendix H Network FLOP counts

Each network presented in our analyses uses a different number of total FLOPs. Additionally, the FLOPs per timestep also differs between them. Here, we report this data as two tables: a) the raw number of FLOPs used by each network at each timestep (Table H1), and b) the number of FLOPs added per timestep in each network as a percentage of the total number of FLOPs (Table H2). Additionally, we tested if the gain in accuracy per timestep is correlated with the gain in FLOPs per timestep and observed no significant correlation as shown in Figure H7.

Network	FLOPs at each timestep (MFLOPs)					
	$t = 0$	$t = 1$	$t = 2$	$t = 3$	$t = 4$	$t = 5$
CNet-parallel	0	217.99	314.86	406.37	496.79	580.70
CNet-serial	0	214.99	312.17	405.04	490.02	580.45
SCAN	0	190.89	308.34	437.46	558.04	627.72
ConvRNN	0	145.02	247.11	338.23	439.05	531.98
MSDNet	0	79.24	159.93	196.44	247.63	317.96

Table H1 FLOPs at each timestep for different networks

Network	Total MFLOPs	FLOPs per timestep (% of total)				
		t=0→1	t=1→2	t=2→3	t=3→4	t=4→5
CNet-parallel	580.70	37.54%	16.68%	15.76%	15.57%	14.46%
CNet-serial	580.45	37.04%	16.74%	16.00%	14.64%	15.58%
SCAN	627.72	30.41%	18.71%	20.57%	19.21%	11.10%
ConvRNN	531.98	27.26%	19.19%	17.13%	18.95%	17.48%
MSDNet	317.96	24.92%	25.38%	11.48%	16.10%	22.13%

Table H2 Cross-network comparison of total MFLOPs and FLOPs incremented per timestep

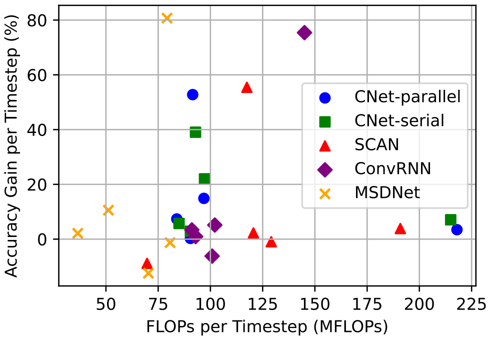

Fig. H7 *Gain in accuracy per timestep vs Increment in FLOPs per timestep.* We observed no significant correlation indicating that the gain in accuracy per timestep cannot be explained by the amount of FLOPs used between timesteps.

Appendix I Compute resources

In order to train and test models for all our experiments, we used resources from an internal cluster at New York University. All networks were trained using 1-2 NVIDIA Tesla V100 GPUs requiring less than 100 GB of memory. Training time for all networks was under 2 days. For each run of inference, we used either a single NVIDIA GeForce GTX 1080 Ti or Tesla V100 GPU.

Appendix J Datasheet for human data

Data was collected for a timed object recognition task at varying levels of difficulty by having a rigid time regime and perturbations. Forced-choice responses of a test subject were recorded for different levels of perturbation in a timed setting.

J.1 Motivation

The core of everyday tasks like reading and driving is active object recognition. Attempts to model such tasks are currently stymied by the inability to incorporate time. People show a flexible tradeoff between speed and accuracy and this tradeoff is a crucial human skill. Deep neural networks have emerged as promising candidates for predicting peak human object recognition performance and neural activity. However, modeling the temporal dimension i.e., the speed-accuracy tradeoff (SAT), is essential for them to serve as useful computational models for how humans recognize objects. To this end, we here present the first large-scale (148 observers, 4 neural networks, 8 tasks) dataset of the speed-accuracy tradeoff (SAT) in recognizing ImageNet images.

The dataset was created at the Pelli Lab, Department of Psychology, New York University, via Lab.js for survey creation, JATOS for hosting and Amazon Mturk for crowdsourced data collection.

J.2 Composition

Dataset is divided into 3 sets based on what perturbation was used while collecting them. Table J3 provides a summary of dataset statistics.

**Perturbations**	**Participants**	**Avg. Compl. (min)**	**#Trials**
Color/Gray	58	43.24	1100
Noise	45	41.32	1100
Blur	45	39.29	1100

Table J3 *Summary of data collected via MTurk.*

J.2.1 Demographic information

We collect performance statistics from 148 observers (88 male, 59 female, 1 non-binary) whose ages ranged from 26 to 70 years, and who agreed to participate in an hour-long session. Each observer had normal or corrected-to-normal vision.

J.2.2 Description of raw data

Information collected via surveys is provided as JSON text files consisting of different fields important for measuring a speed-accuracy trade-off in an observer. The format of the data collection method can be found, in more detail, at lab.js: https://lab.js.org/. Important fields utilize to plot the speed-accuracy trade-off for an observer are:

- **url**: The first row of this column contains the 'srid', a unique ID for each participant in the experiment.
- **order**: +1 indicates RT blocks were presented in ascending order and -1 means they were presented in descending order.
- **sender**: Name of the page presented to the observer.
- **filename**: Filename of image presented.
- **mode**: Value of perturbation applied to the image. 'c' for color, 'g' for grayscale and noise/blur value for noise and blur experiments.
- **correctResponse**: Ground-truth category name of presented image.
- **response**: Observer's category response.
- **duration**: Reaction time of observer response.
- **correct**: 1.0 if observer response was correct, else 0.0.

To understand how to process the raw data collected via surveys, please look at: https://github.com/ajaysub110/satbench/tree/main/human_data_analysis.

J.2.3 Dependency

This dataset of human observers was possible because of the public availability of the ImageNet dataset (Russakovsky et al., 2015) and 16-class ImageNet subset (Geirhos et al., 2018). There are no restrictions on using it for research purposes. Examples of perturbations on ImageNet images can be found in the main manuscript. For the ImageNet and 16-class ImageNet licenses, please visit: https://www.image-net.org/index.php and https://github.com/rgeirhos/generalisation-humans-DNNs.

J.2.4 Participants

We collect age and gender from participants taking the survey. No other information is collected. This dataset cannot be used to calculate any sub-populations, or identify individuals directly or indirectly.

J.3 Collection process

To collect data, we used lab.js (Henninger, Shevchenko, Mertens, Kieslich, & Hilbig, 2020) to design our surveys, JATOS (Lange et al., 2015) to host them, and MTurk to pay participants 20$ per hours for their efforts, with a total of $3860 with all fees.

J.3.1 Survey design

Prior to presenting the stimuli, a sample of 1,100 images was taken randomly from the ImageNet validation dataset and different perturbations were added to create a sample set which was added to the survey.

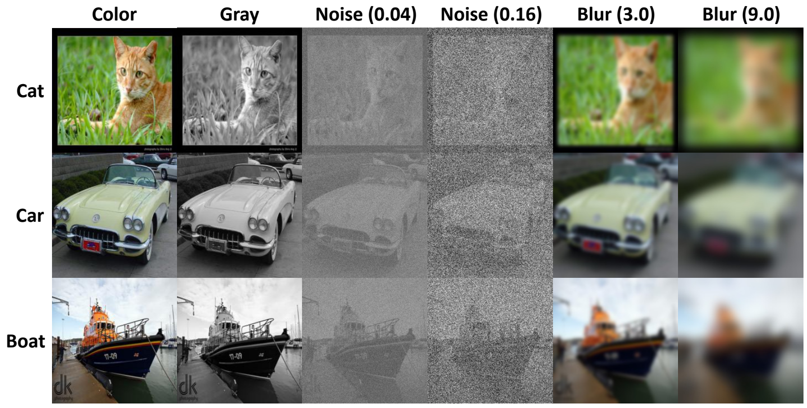

Fig. J8 *Example images from the ImageNet dataset (Russakovsky et al., 2015) along with visualizations of image perturbations considered for human subject experiments – grayscale conversion, image blurring and noise.* Numbers in parentheses correspond to standard deviations for 0-mean Gaussian distributions. Replicated from main paper.

The stimuli were presented via JATOS survey via worker links to each observer. A standard IRB approved (IRB-FY2016-404) consent form was signed before collecting the data by each observer, and demographic information (age and gender) was collected. For different perturbations, observers were given specific instructions to complete the survey.

Prior to the study, subjects were instructed to click on the buttons corresponding to each of the 16 object categories: airplane, bear, bicycle, bird, boat, bottle, car, cat, chair, clock, dog, elephant, keyboard, knife, oven and truck. They also had a training run where they were asked to categorize 50 images.

Stimuli images were scaled to 400x400 pixels for optimal viewing. The survey was designed on five fixed viewing conditions (blocks) of 500 ms, 900 ms, 1100 ms, 1300 ms and 1500 ms with a tolerance of 100 ms each. Outside of these tolerance values, trials were discarded.

For color, noise and blur surveys, each time condition block consisted of 210 trials plus training (1100 trials in total). At the end of the time-limit for a trial, a beep sounded within 60 ms of which the observer had to enter their category decision via virtual button-click after which feedback was given: if they were quick, slow or perfect while clicking the button.

J.4 Time for collection

Designing of a survey took around 1 month. Using MTurk for getting data was faster and data for 33 observers was done within 1.5 months. An IRB-approved form was signed before the start of each survey and a participant had the right to withdraw from the survey at any time.

J.4.1 Hosting the survey

We used JATOS to host and deploy surveys created using lab.js. Hosted surveys can be accessed at:

- Noise: http://64.225.11.86/publix/171/start?batchId=174&generalMultiple
- Blur: http://64.225.11.86/publix/170/start?batchId=173&generalMultiple
- Color/Gray: http://64.225.11.86/publix/167/start?batchId=170&generalMultiple

J.5 Preprocessing

Dataset was collected in the form of surveys and has information related to reaction time and noise. The Jupyter notebooks provided showcase how to process the dataset and create a benchmark for modeling human reaction time. Each psychometric function or data collected from a single observer is in the form of a JSON text file which can be imported as a dataframe using pandas (pandas development team, 2020; Wes McKinney, 2010) in python language. You can checkout how to process data here: https://github.com/ajaysub110/satbench/tree/main/human_data_analysis.

J.6 Uses

The main purpose of this dataset is to provide a benchmark for models exhibiting anytime prediction ability or the ability to effectively trade-off speed and accuracy. Our work compares neural networks with humans on the speed-accuracy trade-off (SAT) task of object recognition and is a fundamental step to understanding various public health issues, such as dyslexia. Possible applications also include object detection in resource-constrained devices and self-driving cars.

J.6.1 Visualizations

Visualizations of observer SAT curves across all conditions are shown in Figure B2. Further analysis can be done by using notebooks provided in https://github.com/ajaysub110/satbench/tree/main/human_data_analysis.

Appendix K Additional dataset information

K.1 Accessing Our Dataset

Our dataset is publicly available at https://osf.io/2cpmb/ and along with visualization notebooks and a detailed description of its contents at https://github.com/ajaysub110/satbench. We guarantee that all results and observations from the paper can be replicated using the code and data available in the repository.

K.2 Author statement

We confirm that we will abide by the rules of the Creative Commons (CC) License and will take responsibility for any violation of rights.
